# Non-fluoroscopic Techniques for Catheter Ablation of Typical Atrioventricular Nodal Re-entry Tachycardia in a Pediatric Patient with Atypical Venous Anatomy

**DOI:** 10.19102/icrm.2022.130802

**Published:** 2022-08-15

**Authors:** Soham Dasgupta, Christopher Johnsrude

**Affiliations:** ^1^Division of Pediatric and Adult Congenital Electrophysiology, Department of Pediatrics, Norton Children’s Hospital, University of Louisville, Louisville, KY, USA

**Keywords:** Ablation, congenital heart disease, fluoroscopy reduction, supraventricular tachycardia

## Abstract

Catheter ablation of the slow pathway to the atrioventricular node is generally a safe and effective treatment for atrioventricular nodal re-entry tachycardia (AVNRT). However, congenital anomalies of the inferior vena cava and superior draining veins can limit traditional catheter approaches to the right heart from femoral venous access and distort local anatomy within the triangle of Koch, necessitating alternative electrophysiology (EP) mapping and ablation strategies. Despite the widespread availability of non-fluoroscopic 3-dimensional imaging systems, many providers still rely on venography to describe unusual cardiovascular anatomy and fluoroscopy to position EP catheters when mapping and ablating the slow pathway. Herein, we report our experience with a pediatric patient with inducible AVNRT and atypical venous anatomy in whom slow pathway ablation was performed successfully without the use of fluoroscopy. In addition, we describe the modification of a novel mapping technique for targeting the slow pathway for ablation.

## Introduction

Atrioventricular (AV) nodal (AVN) re-entry tachycardia (AVNRT) is the most common form of paroxysmal supraventricular tachycardia in adults and adolescents.^1^ Catheter ablation of the right inferior extension of the AVN, the so-called slow pathway, is generally a safe and effective treatment for this arrhythmia. Although electrophysiology (EP) catheters are typically advanced into the right heart from femoral venous access through the inferior vena cava (IVC),^2^ the presence of congenital anomalies of the systemic venous system, especially involving the IVC, can preclude such an approach, necessitating alternative strategies for mapping and ablation. Despite the widespread availability of non-fluoroscopic 3-dimensional (3D) imaging systems to facilitate precise EP mapping of the slow pathway, many centers still rely on fluoroscopy to assist in positioning EP catheters in patients with unusual cardiovascular anatomy. Furthermore, approaches to EP mapping to target the slow pathway continue to evolve with advances in mapping technologies. Herein, we report a case of a 15-year-old girl with unexpected atypical venous anatomy and inducible AVNRT whose venous anatomy and location of the slow pathway to the AVN were determined and in whom catheter ablation performed successfully using a new mapping technique without fluoroscopy.

## Case presentation

**Figure 1:**
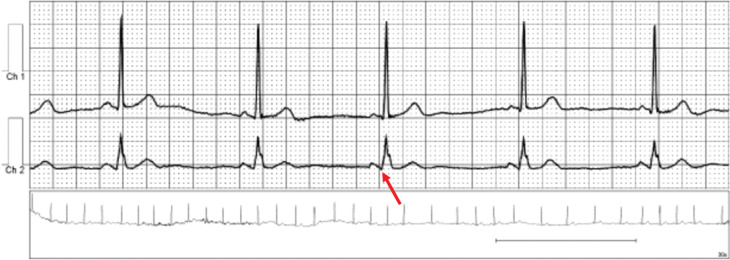
Tracing from a 30-day event recorder suggestive of intermittent ventricular pre-excitation (red arrow).

**Figure 2:**
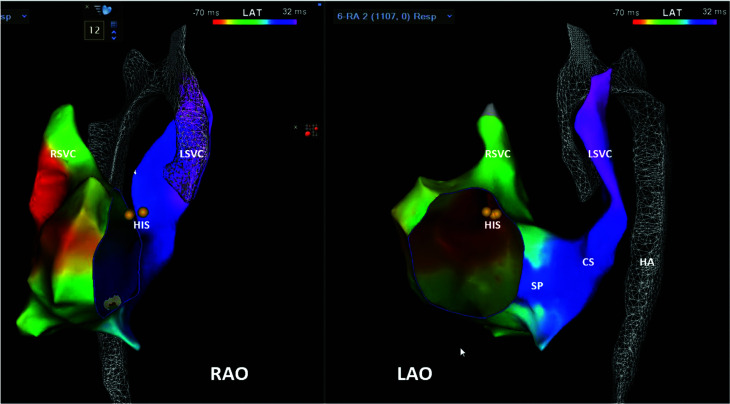
Three-dimensional geometries (right and left anterior oblique views) demonstrating an interrupted inferior vena cava with hemiazygous continuation to a persistent left superior vena cava and dilated coronary sinus. *Abbreviations:* ABL, ablation; CS, coronary sinus; HS, hemiazygous; LAO, left anterior oblique; LSVC, left superior vena cava; RAO, right anterior oblique; RSVC, right superior vena cava; SP, slow pathway.

**Figure 3:**
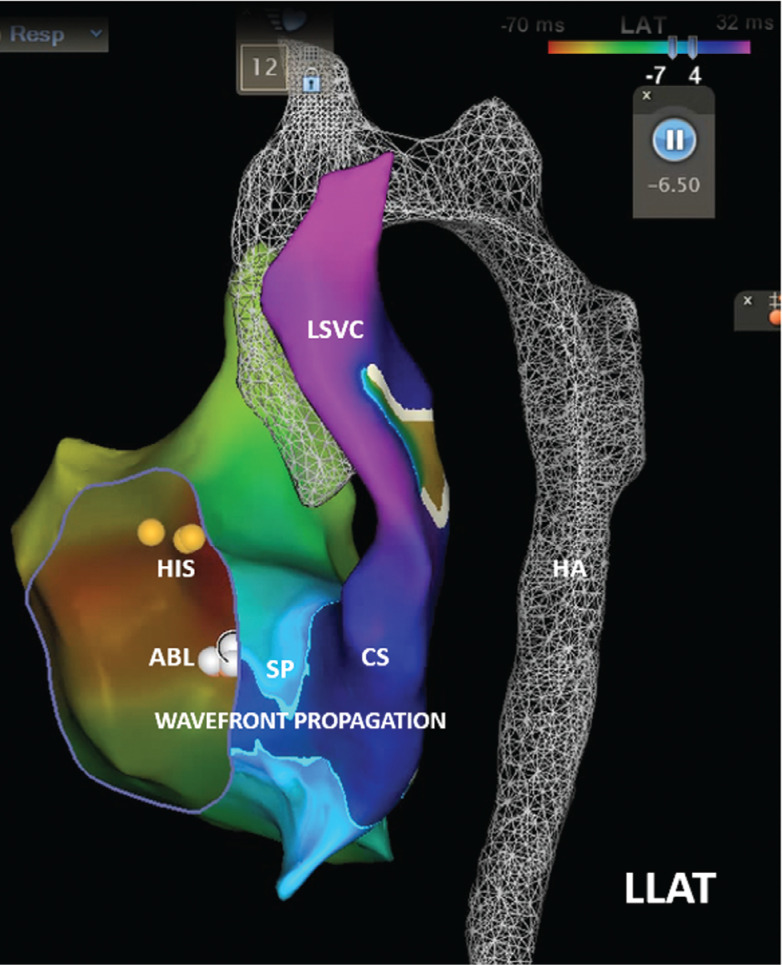
Wavefront propagation map demonstrating a discrete area of abrupt wavefront slowing in the triangle of Koch. *Abbreviations:* CS, coronary sinus; LLAT, left lateral projection; LSVC, left superior vena cava.

A 15-year-old otherwise healthy girl (41 kg) experiencing palpitations while playing volleyball was referred for an EP study and catheter ablation after an asymptomatic 30-day event recorder suggested what might be intermittent ventricular pre-excitation **(Figure 1)**. Her pre-procedural echocardiogram was reportedly unremarkable except for persistent left superior vena cava (LSVC) draining into a dilated coronary sinus (CS). During the EP study, a D-curve Navistar™ mapping/ablation catheter (non-contact force, non-irrigated radiofrequency [RF] ablation catheter; Biosense Webster, Diamond Bar, CA, USA) was advanced fluorolessly via femoral venous access to generate 3D geometries of the right heart, but unexpectedly coursed posterior, leftward, and superior to the heart, consistent with an interrupted IVC and hemiazygos continuation to the LSVC and dilated CS **(Figure 2)**. Creation of adequate 3D geometries and endocardial signal mapping were limited by the circuitous catheter course to the relevant right heart structures. The Navistar™ catheter was therefore deployed via a 7-French (Fr) right internal jugular (RIJ) venous sheath, from which more complete intracardiac geometries were created; a second 5-Fr RIJ sheath was placed to record His-bundle electrograms and record/pace from the right ventricle. To minimize the number of superior venous sheaths and intracardiac EP catheters, a transesophageal EP catheter (TEEP, not shown) was deployed for temporal reference during activation mapping and to provide an additional site for atrial pacing. EP testing demonstrated no evidence of AV accessory pathway conduction but instead demonstrated discrete dual AVN physiology (A–H jump) and inducible typical slow–fast AVNRT. Using the TEEP catheter for reference, dense EP activation and wavefront propagation mapping using a multipolar PentaRay^®^ catheter (Biosense Webster) within the right atrium during sinus rhythm revealed a discrete area of abrupt wavefront slowing in the triangle of Koch and where apparent slow pathway potentials were recorded **(Figure 3 and Video 1)**. This represented the likely position of the slow pathway based on our recent experience using this technology. RF ablation via the RIJ sheath during sinus rhythm resulted in accelerated junctional rhythm during the ablation and elimination of slow pathway conduction (absence of A–H jump) and AVNRT inducibility.

This study was approved by the institutional review board at Norton Children’s Hospital.

## Discussion

To the best of our knowledge, this is the first pediatric case report describing catheter ablation of AVNRT without the use of fluoroscopy in a patient with an interrupted IVC with hemiazygos continuation to an LSVC/dilated CS. Congenital anomalies of the IVC occur in 0.07%–8.7%^3^ of the general population, and interruption of the IVC with azygos continuation occurs in 0.6%–2% of patients with congenital heart disease and <0.3% of patients with otherwise structurally normal hearts.^4^ Interruption of the IVC with hemiazygos continuation to an LSVC, as seen in our patient, is extremely rare.^5^ Persistent LSVC is the most common congenital anomaly of the thoracic venous system with an incidence of 0.3%–0.5% in the adult population.^6,7^ In most cases, a persistent LSVC draining into the right atrium via the CS causes dilation of the CS ostium that can impact 3D EP mapping within a somewhat distorted triangle of Koch,^8^ influence the optimal approach for implanting a transvenous pacemaker or defibrillator, or necessitate modified techniques in patients undergoing cardiac surgery.^9^

In patients with interrupted IVC and azygos/hemiazygos continuation, catheter access to the right heart can be achieved via a femoral venous sheath through the anomalous venous channel, but the circuitous route significantly limits the reach and precise positioning of EP catheters for detailed EP mapping and ablation, as encountered in our case. In this circumstance, alternative access sites such as the RIJ should be considered for performing the procedure. Indeed, prior reports of slow pathway ablation via the RIJ also suggest a potential advantage of this approach, as the shaft of the ablation catheter lies superior to the Eustachian ridge, an occasional impediment to catheter tip positioning at the optimal ablation target when advanced from a femoral venous sheath.^10^

A prior case report described that catheter ablation via the RIJ approach in an adult patient with AVNRT and interrupted IVC with hemiazygos continuation to an LSVC^10^ was ultimately successful, but noted several disadvantages with this approach. The authors reported greater radiation exposure to the operator from close proximity to the image intensifier and an inability to use a side lead shield and difficulty simultaneously manipulating catheters and viewing fluoroscopic images and intracardiac electrograms from unconventional angles.^10^ In addition, the authors performed venography to clarify the anatomy, thereby increasing radiation to the patient and staff. In our case, these concerns were mitigated by (1) creating detailed 3D CARTO^®^ 3 reconstructions of the hemiazygos, LSVC, dilated CS, and portions of the right heart via the anomalous channel from the femoral venous approach; (2) positioning relatively mobile EP recording monitors to improve sight lines when manipulating catheters advanced from the RIJ; (3) supplementing 3D geometry creation and collecting dense endocardial signals in sinus rhythm via a mapping catheter advanced from the RIJ; (4) creating wavefront propagation maps using the CARTO^®^ 3 EML™ software (Biosense Webster); and (5) delivering RF energy at the putative slow pathway via the RIJ approach, all accomplished without fluoroscopy. It is noteworthy that our approach involved 2 sheaths in the RIJ to advance mapping/ablation and diagnostic EP catheters into the right heart and a TEEP catheter for temporal reference so as to limit the need for additional venous sheaths and intracardiac EP catheters. As expected, the RIJ approach facilitated precise and stable catheter positioning, leading to successful slow pathway ablation for AVNRT.

Finally, this case illustrates a novel mapping technique for identifying the precise location of the slow pathway to the AVN in patients with AVNRT. Prior methods using fluoroscopy or 3D imaging systems have involved an anatomical approach based on landmarks of Koch’s triangle,^11^ sometimes incorporating the morphology of local endocardial electrograms^12^ and/or voltage mapping within the posteroseptal space.^13^ Recently, a technique involving mapping of wavefront propagation in the triangle during sinus rhythm was described, where the site of wavefront convergence or “collision” helped to identify the likely location of the slow pathway.^14^ A modification of this latter technique was used here based on our observations using this latter approach in other patients over the last 2 years. Specifically, we identified a site of abrupt slowing of wavefront propagation in the triangle by adjusting the CARTO^®^ 3 early-meets-late lower threshold to display a small island of tissue that was just craniad to the site of wavefront collision (see **Figure 3**); in addition, slow pathway potentials were recorded at this site. As has been our previous experience, RF ablation at that site caused junctional acceleration and successfully eliminated slow pathway conduction and AVNRT inducibility in this patient.

## Conclusion

Congenital vascular anomalies can elude pre-procedural imaging and impose unexpected challenges during catheter-based interventions in the EP laboratory. However, this case shows that clarification of unusual venous anatomy, precise EP mapping, and successful catheter ablation of the slow pathway to the AV node can be readily achieved with current 3D mapping systems and evolving techniques to identify the slow pathway to the AVN, even without the use of fluoroscopy.
